# Dracorhodin perchlorate inhibits osteoclastogenesis through repressing RANKL‐stimulated NFATc1 activity

**DOI:** 10.1111/jcmm.15003

**Published:** 2020-01-21

**Authors:** Yuhao Liu, Ziyi Wang, Chao Ma, Zhenquan Wei, Kai Chen, Chao Wang, Chi Zhou, Leilei Chen, Qingwen Zhang, Zhenqiu Chen, Wei He, Jiake Xu

**Affiliations:** ^1^ Department of Joint Orthopaedic The First Affiliated Hospital Guangzhou University of Chinese Medicine Guangzhou China; ^2^ School of Biomedical Sciences University of Western Australia Perth WA Australia; ^3^ The Lab of Orthopaedics of Chinese Medicine Lingnan Medical Research Center Guangzhou University of Chinese Medicine Guangzhou China; ^4^ The First Clinical Medical College Guangzhou University of Chinese Medicine Guangzhou China; ^5^ Jinshazhou Hospital Guangzhou University of Chinese Medicine Guangzhou China

**Keywords:** dracorhodin perchlorate, nuclear factor of activated T cells 1, osteoclast, osteolysis, receptor‐activated nuclear factor kappa‐B ligand

## Abstract

Osteolytic skeletal disorders are caused by an imbalance in the osteoclast and osteoblast function. Suppressing the differentiation and resorptive function of osteoclast is a key strategy for treating osteolytic diseases. Dracorhodin perchlorate (D.P), an active component from dragon blood resin, has been used for facilitating wound healing and anti‐cancer treatments. In this study, we determined the effect of D.P on osteoclast differentiation and function. We have found that D.P inhibited RANKL‐induced osteoclast formation and resorbed pits of hydroxyapatite‐coated plate in a dose‐dependent manner. D.P also disrupted the formation of intact actin‐rich podosome structures in mature osteoclasts and inhibited osteoclast‐specific gene and protein expressions. Further, D.P was able to suppress RANKL‐activated JNK, NF‐κB and Ca^2+^ signalling pathways and reduces the expression level of NFATc1 as well as the nucleus translocation of NFATc1. Overall, these results indicated a potential therapeutic effect of D.P on osteoclast‐related conditions.

## 1 INTRODUCTION

Dracorhodin perchlorate (D.P), derived from dracorhodin, is a major active component of dragon blood (*Sanguis draxonis* or *Daemonorops draco*) resin, a traditional herbal medicine, which was commonly used for the therapy of injuries, such as wounds and fractures.^1^, ^2^, ^3^ D.P has multiple applications in medicine, for example, D.P is found to enhance cutaneous wound healing by promoting fibroblasts proliferation in rats.^4^, ^5^ D.P also exerts strong angiogenic effects, which can be beneficial for wound healing.^6^, ^7^ D.P is found to have an anti‐cancer function by inducing apoptosis in human cancer cells.^8^, ^9^, ^10^, ^11^, ^12^ Reported in some other literatures, D.P has anti‐microbial effects by reducing the production of α‐toxin by *Staphylococcus aureus* 13 and inhibiting the formation of biofilm and virulence factors of *Candida albicans*.^14^ We have confirmed that several compounds extracted from *S draxonis* resin have inhibitory effects on osteoclast differentiation.^15^ However, the effect of D.P on skeletal disorders is not yet determined.

Bone diseases, such as osteoporosis, osteonecrosis and Paget's disease, are all related to the over‐activation of osteoclasts. Osteoclasts, the bone resorptive cells, and osteoblasts, the bone‐forming cells, interact with each other in a way that the skeletal tissue is able to undergo constant remodelling and maintain the daily life of the vertebrates.^16^ However, imbalance of activities between these two types of cells can occur under certain situations, for example, postmenopausal women have higher risks of developing osteoporosis than other population due to oestrogen deficiency.^16^, ^17^ There are limited options of the treatment of osteoporosis which is of huge threat to the patients' well‐being and causing enormous socio‐economic burdens.^18^, ^19^, ^20^

Osteoclasts are originated from the haematopoietic stem cells. The differentiation of osteoclasts from the precursor cells is dependent on two important signalling molecules, macrophage colony‐stimulating factor (M‐CSF) and receptor activated nuclear factor kappa‐B ligand (RANKL).^21^ RANKL belongs to the TNF family, which binds to RANK on the surface of osteoclast precursor cells and mediates the formation and function of osteoclast. Upon binding to RANK, RANKL activates several pathways to initiate the cell differentiation of osteoclast precursors to mature osteoclasts, importantly, MAPK pathway and NF‐κB pathway.^22^, ^23^, ^24^ These two pathways both activate the transcription of nuclear factor of activated T cells 1 (NFATc1), the master transcription factor of osteoclast‐specific genes, such as cathepsin K, TRAcP, calcitonin receptor and c‐Fos.^25^ These proteins all contribute to the formation and function of osteoclast.

In this study, using in vitro experiments, we were able to show the inhibitory effect of D.P on RANKL‐induced osteoclast formation and function. We found this inhibitory effect was mainly caused by impeding the activation of NF‐κB and MAPK pathways, which leads to a reduction of NFATc1 expression. Furthermore, the activation of calcium pathway and NFATc1 was also suppressed by D.P. Our study demonstrated the therapeutic potential of D.P to osteoclast‐related bone diseases.

## 2 MATERIALS AND METHODS

### 2.1 Reagents

Dracorhodin perchlorate (D.P) was obtained from Chengdu Must Bio‐Technology Co., Ltd, and was diluted in DMSO at a concentration of 100 mmol/L for storage, which was further diluted to the working dilutions with PBS. Alpha‐Minimum essential medium (α‐MEM, REF#12571‐063), foetal bovine serum (FBS, REF#16000‐044), DAPI (Cat#D3571), Hoechst 33258 dye (REF#H21491), rhodamine phalloidin (Cat#R415) and ProLong Gold Antifade Mountant (REF#P36970) were commercially available from Thermo Fisher Scientific. MTS kit was obtained from Promega (REF#G358A). Primary antibodies against Integrin β3 (Cat#sc‐6617‐R), MMP9 (Cat#sc‐21733), CTSK (Cat#sc‐48353), β‐actin (Cat#sc‐47778), IκB‐α (Cat#sc‐371), p65 (Cat#sc‐8008), PARP‐1 (Cat#sc‐8007) and NFATc1 (Cat#sc‐7294) were commercially available from Santa Cruz Biotechnology. Primary antibodies against c‐Fos (Cat#2250S), p‐p65 (Cat#3031S), p‐JNK (Cat#9251L) and JNK (Cat#9252L) were purchased from Cell Signalling Technology. Antimouse IgG‐FITC antibody (Cat#F9137), anti‐vinculin antibody (Cat#V9264), anti‐tubulin antibody (Cat#T3526) and recombinant M‐CSF (Cat#M6518) were commercially available from Sigma‐Aldrich. Recombinant GST‐rRANKL was produced and purified as previously described.^26^

### 2.2 Cell culture and osteoclast formation

Bone marrow macrophages (BMMs) were isolated by flushing the bone marrow out of the hindlimbs from C57BL/6J mice (female, 6‐week‐old). The resulting bone marrow cell suspension was then filtered with a 100 μm strainer, resuspended and cultured in α‐MEM with 10% FBS, 100 U/mL penicillin, 100 mg/mL streptomycin (complete α‐MEM). To maintain the growth of BMM culture, 50 ng/mL of M‐CSF was also supplemented into the cell culture media. When the adherent cell became confluent, 6 × 10^3^ BMMs were seeded into 96‐well plates per well and cultured overnight at 37°C in a moist environment with 5% CO_2_. Starting the following day, the BMMs were stimulated with 50 ng/mL of RANKL, with different concentrations of D.P (0, 1, 5, 10, 20, 30 μmol/L) for 5 days before being fixed with 2.5% glutaraldehyde and stained for tartrate‐resistant acid phosphatase (TRAcP) activities. The stained cells were examined and photographed using an inverted light microscope. TRAcP‐positive multinucleated cells (MNCs) were scored as osteoclasts if they had three or more nuclei.

### 2.3 Cytotoxicity assay

6 × 10^3^ BMMs were seeded into 96‐well plates per well. After being incubated overnight, the cells were exposed to different concentrations of D.P (0, 1, 5, 10, 20, 30 μmol/L) for 48 hours. After 48 hours, MTS reagent was added to the cells and incubated. MTS measurement was performed as per manufacturer's instruction.

### 2.4 Hydroxyapatite resorption assay

BMMs were initially plated onto collagen‐coated 6‐well plates (Corning Inc) at a density of 8 × 10^4^ cells per well in complete α‐MEM containing M‐CSF. The cells were then stimulated with 50 ng/mL of RANKL until pre‐mature osteoclasts were formed. Cells were then detached from the plate by cell dissociation solution (Cat#C5914, Sigma‐Aldrich) gently to avoid cell breakage. The harvested pre‐mature osteoclasts were then seeded into strip‐well hydroxyapatite‐coated plates (Corning Inc). The cells were continued to be stimulated by 50 ng/mL of RANKL with or without 30 μmol/L of D.P for 48 hours. After the incubation, the wells were washed with ddH_2_O, followed by that three wells were stained for TRAcP activities and the other three wells were subsequently washed with 10% bleach to get rid of the cells to expose bone resorption areas. The wells were then imaged using an inverted light microscope and analysed using ImageJ software (NIH).

### 2.5 Immunofluorescent microscopy staining and actin belt staining

5 × 10^4^ BMMs were seeded onto glass coverslips (14 mm in diameter) in 24‐well plates per well. The BMMs were then induced to form mature osteoclasts, as described previously, in the presence or absence of D.P (30 μmol/L). The cells were then fixed and permeabilized with 0.1% (v/v) Triton X‐100. After blocking unspecific binding using 3% BSA in PBS, the cells were incubated with anti‐vinculin antibody (1:500) or anti‐NFATc1 antibody (1:500) at 4°C overnight. A secondary antimouse IgG conjugated with FITC was used to produce a fluorescent signal. After the incubation of secondary antibody for 1 hour, the cells were stained with rhodamine phalloidin for 20 minutes. Last, the cells were incubated with DAPI or Hoechst 33258 dye for 10 minutes and mounted to glass slides using the ProLong Gold Antifade Mountant and visualized using NIKON A1Si confocal microscope (Nikon Corporation).

### 2.6 RNA extraction and real‐time PCR

BMMs (1 × 10^5^ cells/well) were cultured and stimulated with RANKL (50 ng/mL) in 6‐well plates with the addition of different concentrations of D.P (0, 20, 30 μmol/L) for 5 days. Total RNA was collected and isolated using TRIzol™ reagent (Thermo Fisher) and PureLink RNA Mini Kit (Thermo Fisher). cDNA was synthesized using 1 μg of RNA with M‐MLV reverse transcriptase (Promega) as per manufacturer's instruction. The resulting cDNA was then used for real‐time PCR with PowerUP™ SYBR Green Master Mix (Thermo Fisher) using an Applied Biosystem ViiATM7 Real‐Time PCR System (Thermo Fisher). The conditions for PCR were as follow: 95°C for 5 minutes, followed by 30 cycles of (95°C for 15 seconds, 60°C for 60 seconds) and a melt curve stage of (95°C for 15 seconds, 60°C for 60 seconds, 95°C for 15 seconds). The specific primers used in this study were as follow, *Acp5* (Forward: 5′‐TGTGGCCATCTTTATG CT‐3′; Reverse: 5′‐GTCATTTCTTTGGGGCTT‐3′), *Nfatc1* (Forward: 5′‐CAACGCCCTGACCACCGATAG‐3′; Reverse: 5′‐ GGCTGCCTTCCGTCTCATAGT‐3′), *Ctr* (Forward: 5′‐TGGTTGAGGTTGTGCCCA‐3′; Reverse: 5′CTCGTGGGTTTGCCTCATC‐3′), *c‐fos* (Forward: 5′‐ GCGAGCAACTGAGAAGAC‐3′; Reverse: 5′‐ TTGAAACCCGAGAACATC‐ 3′), *Ctsk* (Forward: 5′‐GGGAGAAAAACCTGA AGC‐3′; Reverse: 5′ ‐ ATTCTGGGGACTCAGAGC‐3′), *Mmp9* (Forward: 5′‐ CGTGTCTG GAGATTCGACTTGA‐3′; Reverse: 5′‐ TTGGAAACTCACACGCCAGA‐3′), *Hmbs* (Forward: 5′‐ AAGGGCTTTTCTGAGGCACC‐3′; Reverse: 5′‐AGTTGCCCATCTTTCATCACTG‐ 3′), *Hprt1* (Forward: 5′‐TCAGTCAACGGGGGACATAAA‐3′; Reverse: 5′‐ GGGGCTGTACTGCTTAACCAG‐3′). mRNA expression levels were normalized to the mean expression level of *Hprt1* and *Hmbs*. The qPCR results were analysed using ΔΔCT method.

### 2.7 Western blot

For the study of osteoclast‐specific protein expression, the BMMs were seeded into 6‐well plates at a density of 1 × 10^5^ cells/well and stimulated with RANKL (50 ng/mL) and in the presence or absence of D.P (30 μmol/L) for 0, 1, 3 and 5 days separately. For the examination of signalling pathway proteins, the BMMs were seeded into 6‐well plates at a density of 2.5 × 10^5^ cells/well and cultured overnight. The cells were then starved for 1 hour and pre‐treated with D.P (30 μmol/L) for 1 hour followed by stimulation of RANKL (50 ng/mL) for indicated periods. After the stimulation, the cells were incubated with RIPA buffer on ice to obtain the total proteins. The proteins were then separated by SDS‐PAGE and transferred to a nitrocellulose membrane (GE Healthcare). After being blocked in 5% skim milk, the membranes were probed with different primary antibodies at 4°C for overnight. The membranes were then washed in TBS‐Tween and incubated with horseradish peroxidase (HRP) conjugated secondary antibodies at room temperature for 1 hour. The protein bands were visualized using enhanced chemiluminescence substrate (PerkinElmer) and an Image‐quant LAS 4000 system (GE Healthcare). The intensities of bands were measured and analysed using ImageJ software.

### 2.8 Nuclear and cytoplasmic extraction

BMMs were seeded at a density of 1 × 10^5^ cells/well in 6‐well plates. The cells were then stimulated with 50 ng/mL RANKL with or without D.P (30 μmol/L) for 5 days. The cells were trypsinized and lysed using the NE‐PER™ Nuclear and Cytoplasmic Extraction Reagents (Thermo Fisher) as per manufacturers' instructions. The cytoplasmic and nuclear extracts were examined using Western blot. Antibodies against PARP‐1 and tubulin were used as loading controls for nuclear component and cytoplasmic component of the cell, respectively.

### 2.9 Calcium oscillation

BMMs were seeded into 48‐well plates at a density of 3 × 10^4^ cells/well with complete α‐MEM containing 50 ng/mL of M‐CSF. After overnight incubation, the cells were starved for 1 hour and pre‐treated with D.P (30 μmol/L) before the stimulation with RANKL (50 ng/mL). Calcium oscillation was observed by staining the cells with 4 μg/mL of Fluo‐4 AM (Thermo Fisher). The Fluo‐4 AM was dissolved by 20% (w/v) Pluronic F127 (Sigma‐Aldrich) in DMSO and further diluted to the working concentration by Hanks' Balanced Salt Solution (Sigma‐Aldrich) containing 2% FBS and 1 mmol/L probenecid (Sigma‐Aldrich) and then was added to the cells. The cells were observed, and the images were taken using a Nikon inverted fluorescent microscope (Nikon Corporation). Three random fields of view were chosen and filmed for a total of 3 minutes. To analyse the results, 20 cells from each field of view were selected (in total, 60 cells for each well and 180 cells for each group). The frequency of calcium oscillation was measured using the NIS Element software (Nikon Corporation).

### 2.10 Luciferase reporter assay

RAW264.7 cells were stably transfected with an NFATc1 luciferase reporter construct, pGL4.30 [luc2P/NFAT‐RE/Hygro] (Promega). For luciferase reporter assay, 1.5 × 10^4^ cells/well were seeded into 48‐well plates. After overnight incubation, the cells were serum‐starved for 2 hours and pre‐treated with different concentrations of D.P for 1 hour. Then, the cells were stimulated with 50 ng/mL RANKL for 24 hours. When the time‐point was achieved, the cells were lysed, and luciferase reporter assay was performed using Luciferase Assay System (Promega) as per manufacturer's instructions.

### 2.11 Statistical analysis

All experiments were performed with at least 3 replicates. All data in the study were presented as mean ± standard deviation. Statistical analyses were all performed using GraphPad Prism 7 software. For the comparison of two sets of data, Student's *t* tests with assumption of equal variance were used, and for the comparison among 3 or more groups of data, one‐way ANOVA tests were used with Dunnett's test for multiple comparisons between the groups. A *P*‐value ≤ .05 between two groups was considered to be statistically significant (95% confidence interval).

## 3 RESULTS

### 3.1 D.P inhibited RANKL‐induced osteoclastogenesis in vitro

**Figure 1:**
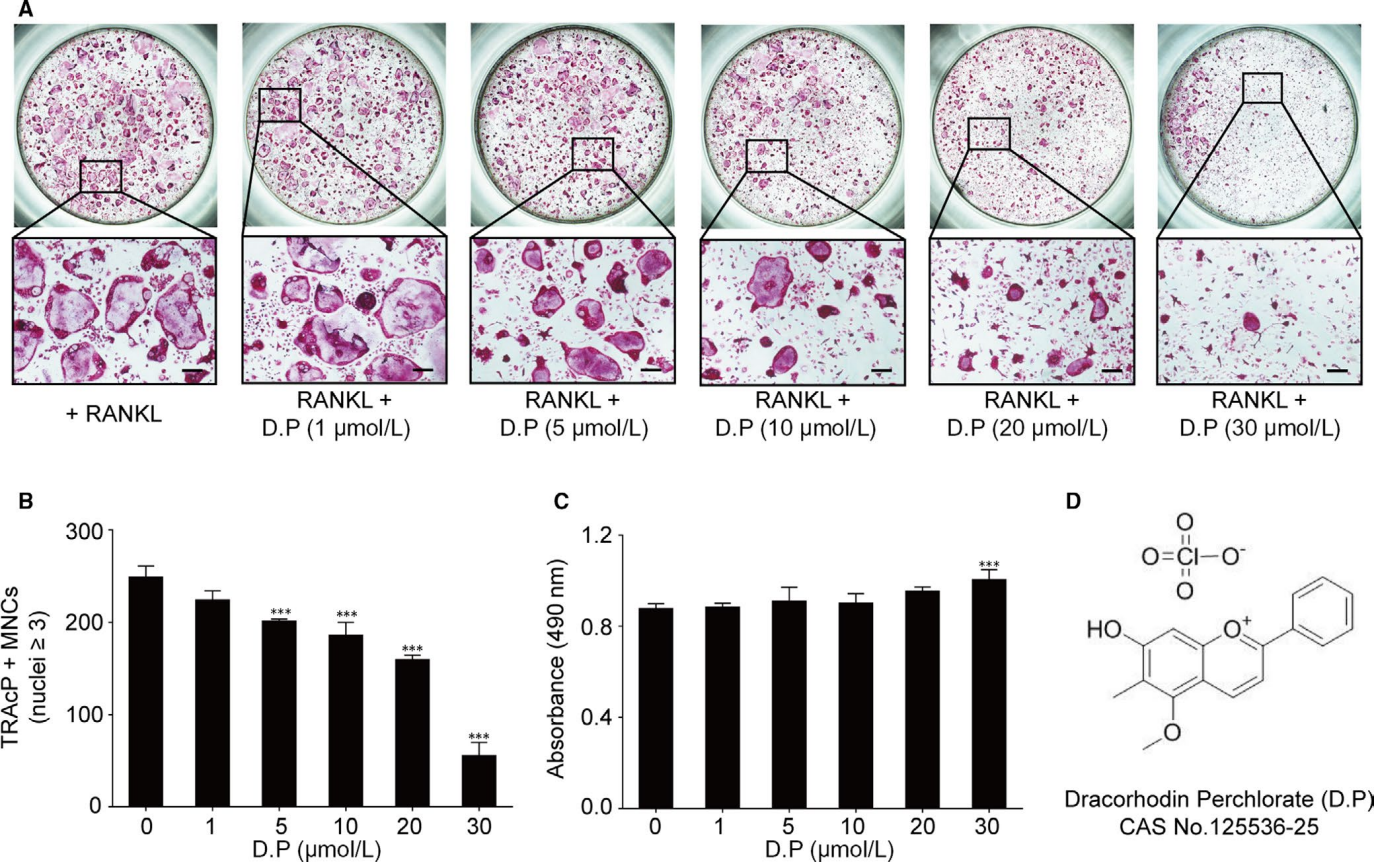
Dracorhodin perchlorate (D.P) suppressed RANKL‐induced osteoclast formation in vitro. (A) Microscopic images of the osteoclast cultures treated with different concentrations of D.P for 5 days (Scale bar = 200 μm). (B) Quantification of TRAcP positive, multinucleated cells (MNCs) in the osteoclast cultures after the treatment of D.P for 5 days. (C) MTS assay of the BMMs stimulated with different concentrations of D.P (D) Chemical structure of D.P. n = 3; ***P < .001

The chemical structure of D.P was shown in Figure 1D. To determine the effect of D.P on RANKL‐induced osteoclast formation, BMMs were seeded onto 96‐well plates and RANKL was then added with or without the presence of DP It was observed in the RANKL control group that TRAcP‐positive multinucleated cells appeared after the stimulation with RANKL. However, with the increasing concentrations of D.P, both the size and the number of multinucleated cell were reduced by the treatment of D.P in a dose‐dependent manner (Figure 1A,B). To eliminate any potential toxicity effects of D.P to BMMs, MTS assay was performed. It was found that D.P had no significant cytotoxic effect on BMMs within the concentration range, used in our study (Figure 1C).

### 3.2 D.P attenuated RANKL‐induced F‐actin belt formation and osteoclastic hydroxyapatite resorption in vitro

**Figure 2:**
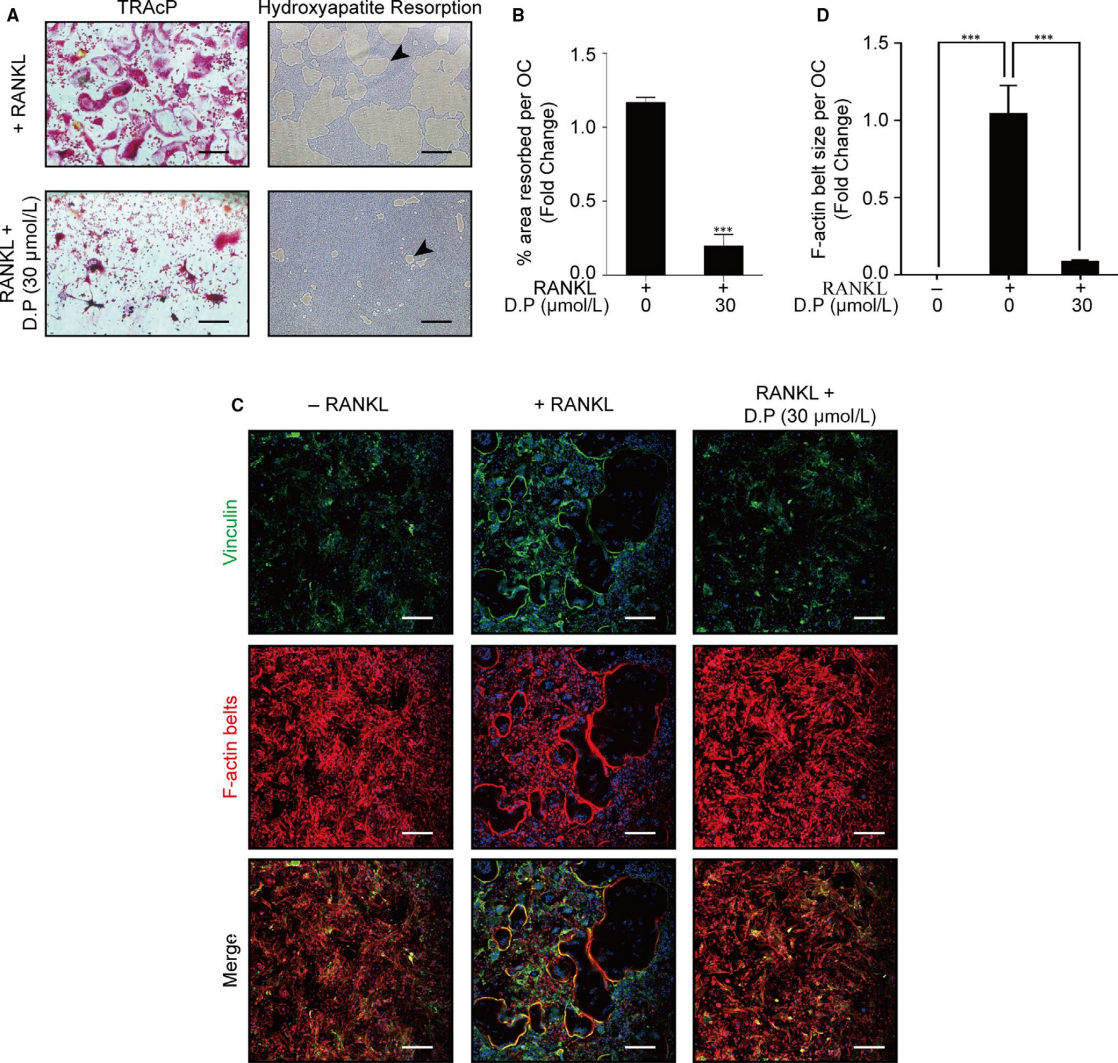
D.P supressed the formation of F‐actin belt and hydroxyapatite resorption. (A) Representative images of the TRAcP staining and resorption on hydroxyapatite‐coated plates (Scale bar = 200 μm). (B) Statistical analysis of the area of hydroxyapatite resorbed per osteoclast. (C) Representative images of immunofluorescent microscopy showing the intact podosome belts disrupted by the addition of D.P. Vinculin (green), F‐actin (red) and nuclei (blue) were co‐stained and observed. (D) Quantification of the size of podosome belt per osteoclast demonstrated as the fold change relative to the podosome size of RANKL control group. n = 3; ***P < .001

Hydroxyapatite‐coated plates were used to examine the effect of D.P on osteoclast function. As the result, the RANKL control group presented numerous TRAcP‐positive multinucleated osteoclasts and the resorption area of the hydroxyapatite was highlighted by the arrow in the figure. In contrast, the intervention of D.P resulted in the decrease of the resorption area. (Figure 2A,B) These results indicated the inhibitory effects of D.P on RANKL‐induced resorptive functions of osteoclasts.

Immunofluorescent imagings with anti‐vinculin antibody and rhodamine phalloidin were used to visualize the podosomes around the cells. It was shown by the results that RANKL induction in BMMs resulted in multinucleated cells surrounded by well‐defined podosome structures with F‐actin belts. Addition of D.P, on the contrary, caused a reduction of the formation of multinucleated cells and F‐actin belts (Figure 2C,D).

### 3.3 D.P suppressed the expression of osteoclast‐specific genes and proteins

**Figure 3:**
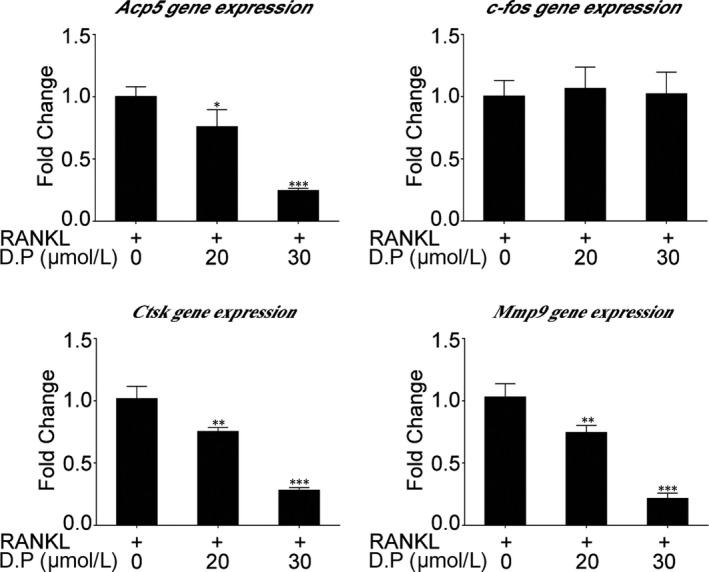
D.P inhibited RANKL‐induced osteoclast‐specific gene expression. Real‐time PCR was utilized to determine the expression levels of osteoclast‐specific genes, *Acp5*, *c‐fos*, *Ctsk* and *Mmp9*. Expression levels were normalized to the mean of *Hprt1* and *Hmbs*. Data were presented as fold changes relative to RANKL control group. n = 3; *P < .05, **P < .01, ***P < .001

Real‐time quantitative PCR was used to determine the effect of D.P on RANKL‐induced expressions of osteoclast‐specific genes. Concluding the results, the expressions of *Acp5*, *Ctsk* and *Mmp9* induced by RANKL, after 5 days of culture, were inhibited by the addition of D.P in a dose‐dependent manner (Figure 3).

**Figure 4:**
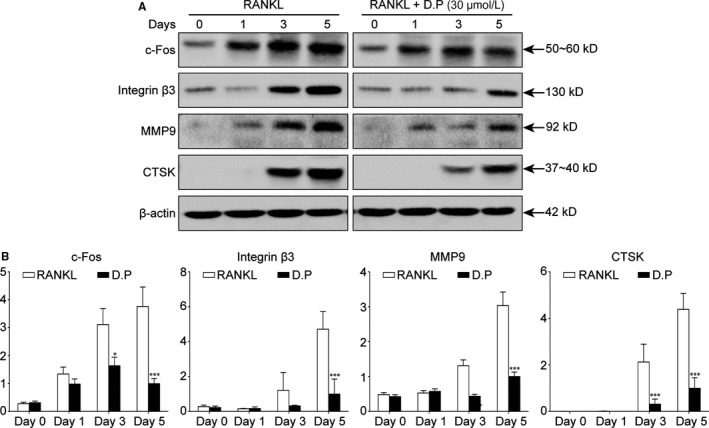
D.P supressed osteoclast marker protein expression. (A) Representative images of Western blot on the effects of D.P on osteoclast‐specific proteins during osteoclastogenesis, c‐Fos, Integrin β3, MMP9 and CTSK. β‐actin was used as a loading control. (B) Quantitative analysis of the band intensities relative to β‐actin intensity. n = 3; *P < .05, ***P < .001

Western blot was utilized to examine the expression levels of osteoclast‐specific proteins. Although the genetic expression level of *c‐fos* was not interfered by D.P, the protein expression of c‐Fos was suppressed. Other osteoclast‐specific proteins had an increase in expression level upon the induction by RANKL during the 5‐day culture, but these enhanced expressions of Integrin β3, MMP9 and CTSK were decreased by the addition of D.P (Figure 4). These results were in line with osteoclast formation assay and hydroxyapatite resorption assay.

### 3.4 D.P suppressed the activation of JNK and p65

**Figure 5:**
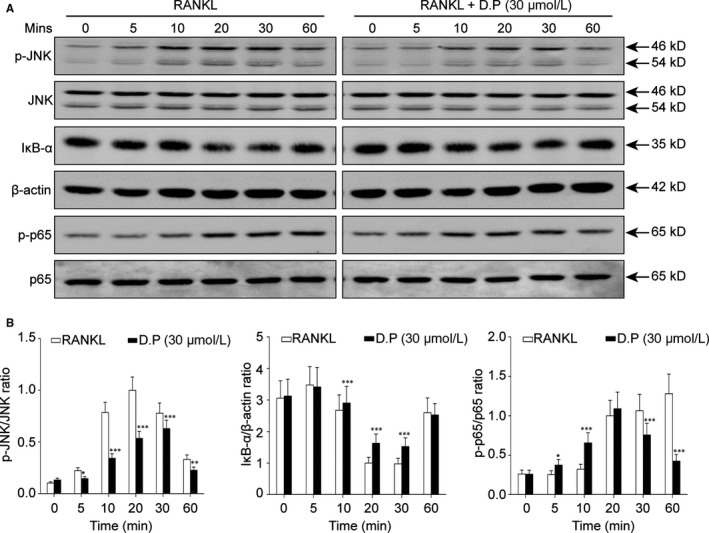
D.P supressed the activation of JNK and NF‐κB by RANKL. The BMMs were stimulated with RANKL for various periods of time with or without D.P. The total protein was examined by Western blot. n = 3; *P < .05, **P < .01, ***P < .001

To elucidate the suppression mechanism of D.P on RANKL‐induced osteoclast formation and function, Western blot was used to examine the activation of some key signalling proteins. According to the results, D.P inhibited the phosphorylation of JNK by RANKL stimulation in BMMs. It was also observed that D.P inhibited the degradation of IκB‐α, and the phosphorylation of p65 subunit of NF‐κB complex mediated by RANKL, indicating an inhibitory effect of D.P on the activation of NF‐κB signalling pathway (Figure 5).

### 3.5 D.P suppressed the activation of NFATc1 by RANKL

**Figure 6:**
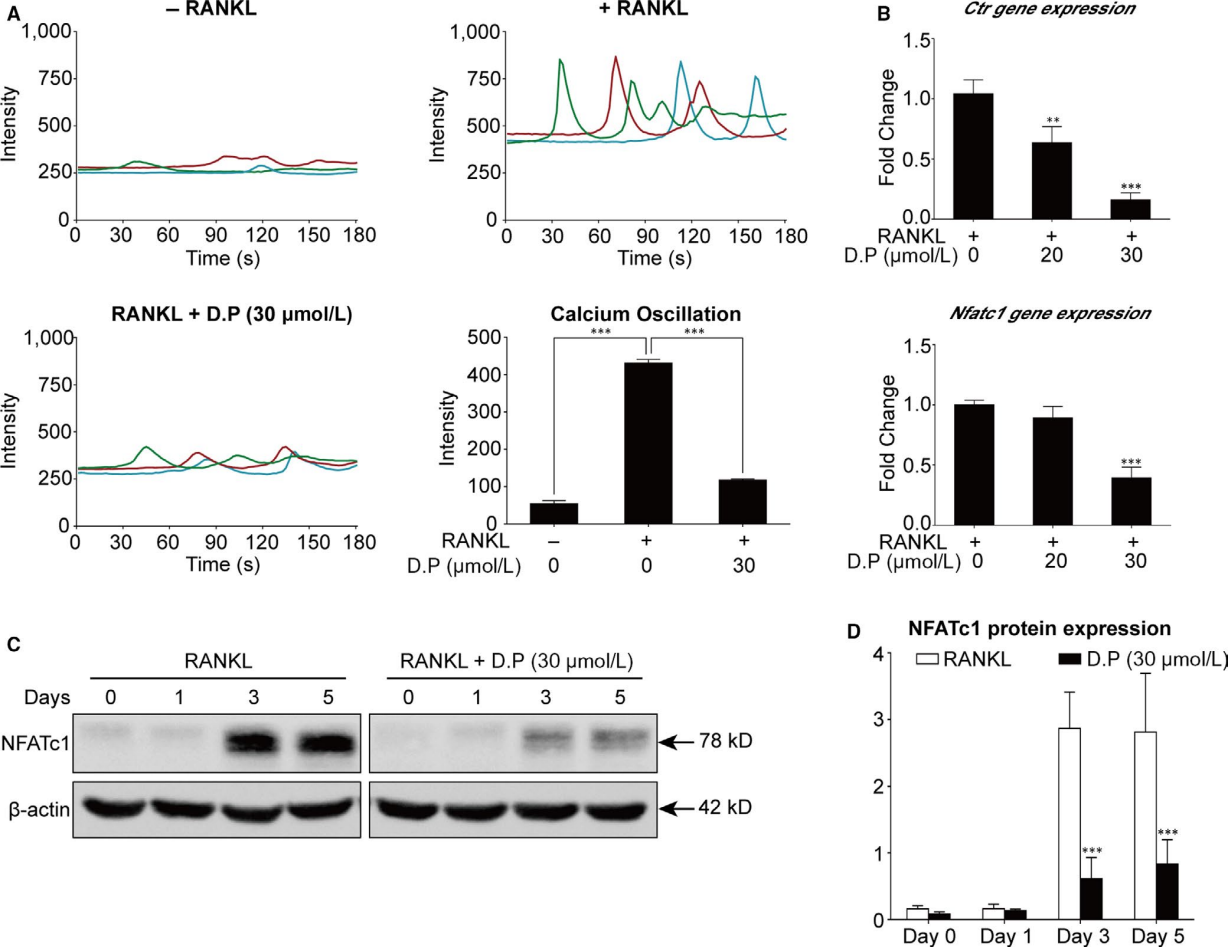
D.P inhibited the NFATc1 expression. (A) Representative traces and quantitative analysis of the calcium oscillation intensity. The results were presented as calcium oscillation frequency. (B) Gene expression levels of *Ctr* and *Nfatc1* were determined using qPCR. The expression levels were normalized to the average expression of *Hprt1* and *Hmbs* and presented as fold changes relative to the RANKL control group. (C) Representative Western blot images of NFATc1 protein level after stimulation with RANKL for 5 days, in the presence or absence of D.P. β‐actin was used as loading control. (D) Quantitative analysis of the NFATc1 protein expression levels relative to the RANKL control group. n = 3; **P < .01, ***P < .001

**Figure 7:**
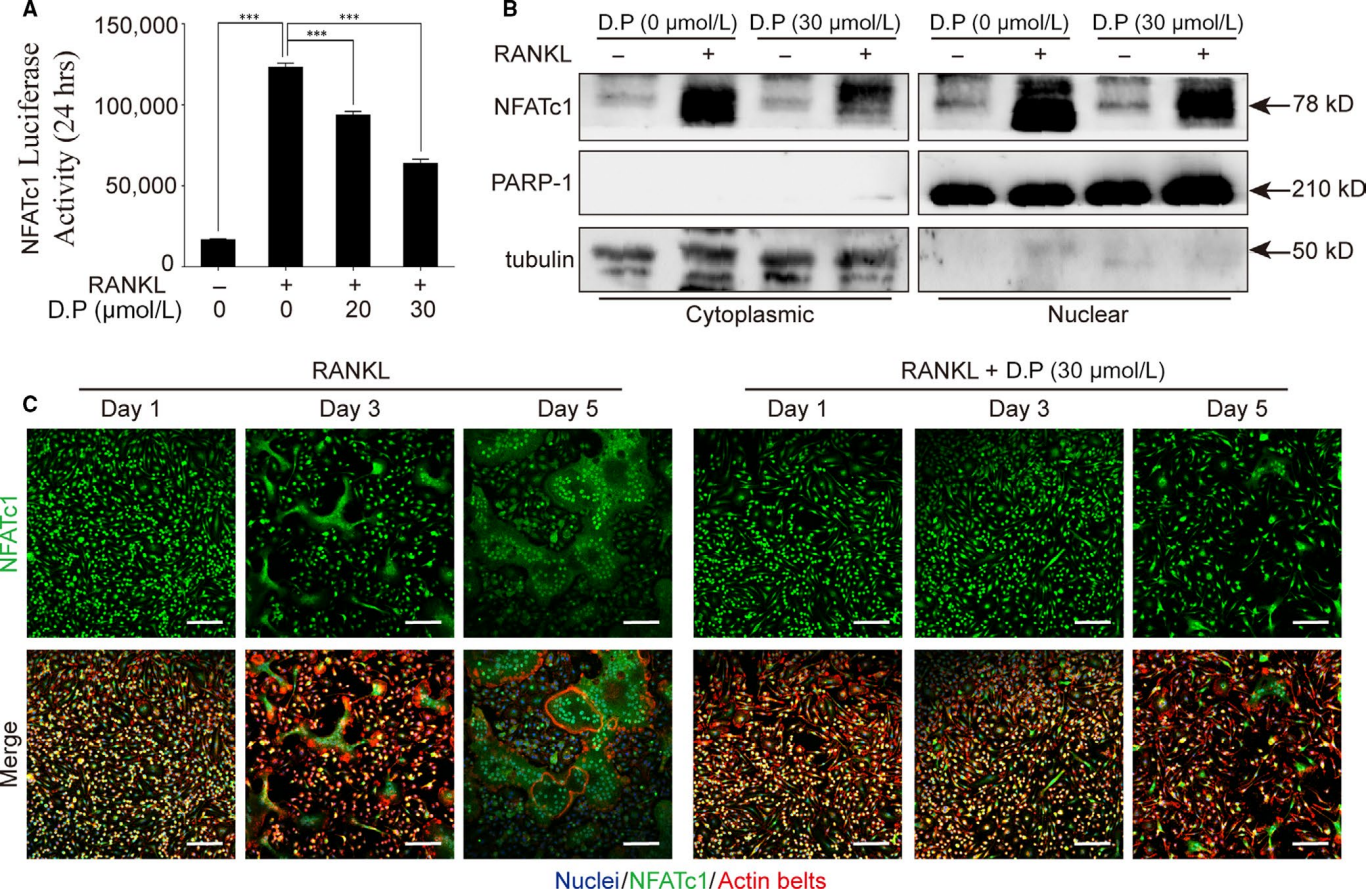
D.P inhibited NFATc1 activity and translocation to nuclei. (A) NFATc1 activity was detected using a luciferase reporter gene assay. n = 3; ***P < .001. (B) Representative images of Western blot to determine NFATc1 translocation after stimulation with RANKL for 5 days in the presence or absence of D.P. PARP‐1 and tubulin were used as cytoplasmic and nuclear loading controls, respectively. (C) Representative images of the immunofluorescent microscopy co‐stained for NFATc1 (green), actin belts (red) and nuclei (blue) (Scale bar = 100 μm)

To examine the effect of D.P on Ca^2+^ signalling pathway in the RANKL‐induced osteoclastogenesis, calcium oscillation assay was performed. Shown by the results, the intensity of calcium oscillation was increased by RANKL, but D.P strongly suppressed the RANKL‐induced calcium oscillation (Figure 6A). Further to the calcium oscillation, it was found by real‐time PCR that the gene expression levels of *Ctr* and *Nfatc1* during RANKL‐induced osteoclastogenesis were also suppressed by D.P (Figure 6B). The protein level of NFATc1 was determined using Western blot. The increased expression of NFATc1 protein induced by RANKL was inhibited by D.P (Figure 6C,D). Furthermore, we determined the attenuation of the NFATc1 signalling pathway by examining the trans‐nuclear movement of NFATc1 using Western blot and immunofluorescent staining of nuclear and cytoplasmic proteins. It was seen that the NFATc1 protein was translocated into the nucleus upon RANKL stimulation; however, the translocation of NFATc1 was inhibited by D.P (Figure 7B,C). We also examined the transcriptional activity of NFATc1 using a luciferase reporter assay, thereafter. It was shown that the activation of NFATc1 by RANKL was inhibited by D.P (Figure 7A). Concluding the results, NFATc1 expression and activity were both inhibited by D.P.

## 4 DISCUSSION

Over‐activation of osteoclast function is one of the mechanisms causing osteolytic diseases, such as osteoporosis, osteonecrosis and Paget's disease.^20^, ^27^, ^28^, ^29^ Current treatments of these diseases are limited, expensive and would cause various side effects.^30^ Natural compounds, found in traditional medicine, have good therapeutic potentials with fewer side effects. Therefore, determining the therapeutic effects of various natural compounds on osteoclast‐related diseases could be beneficial. D.P, as mentioned in the previous content, has been used in the treatment of different diseases. In this study, we have demonstrated its inhibitory effects on osteoclast formation and function.

As a result, we found that D.P was able to inhibit RANKL‐induced osteoclastogenesis in vitro, in a dose‐dependent manner without causing any toxicity effects on the BMMs at any effective concentrations. By immunofluorescent microscopy, we found that the D.P‐treated cells failed to form intact actin‐rich podosomes after the stimulation by RANKL for 5 days, which is essential for the resorptive function of osteoclast.^31^ This result is consistent with our results that the resorptive function of mature osteoclast was affected by D.P. These results suggest that D.P might serve as a potential therapeutic agent for osteoclast‐related diseases. However, the effect of D.P on osteolytic diseases should be further determined using appropriate animal models.

D.P was able to suppress the activation of NF‐κB pathway by inhibiting the degradation of IκB‐α and the phosphorylation of the p65 subunit of the NF‐κB complex. NF‐κB pathway is one of the major signalling events stimulated by RANKL during osteoclastogenesis.^32^ Binding of RANKL to RANK causes the degradation of IκB‐α and subsequent phosphorylation of the NF‐κB p65 subunit, which will then translocate into the nucleus and initiate the expressions of the master transcription factors of osteoclast‐specific genes. Our results demonstrated the inhibitory effect of D.P on the activation of NF‐κB pathway, similar to the previous findings in other cell types.^12^

In addition, D.P suppressed the phosphorylation of JNK, which is a MAPK, downstream of TRAF6. Activation of JNK is critical to the survival of osteoclast.^33^ Reduction of the phosphorylation of JNK was observed by Western blot, and this result was different with the result observed in previous study of other cell type,^34^ where the JNK activity was promoted by D.P. However, this difference in the effect of D.P on the JNK pathway could be a result from the different cell types used in the experiments.

**Figure 8:**
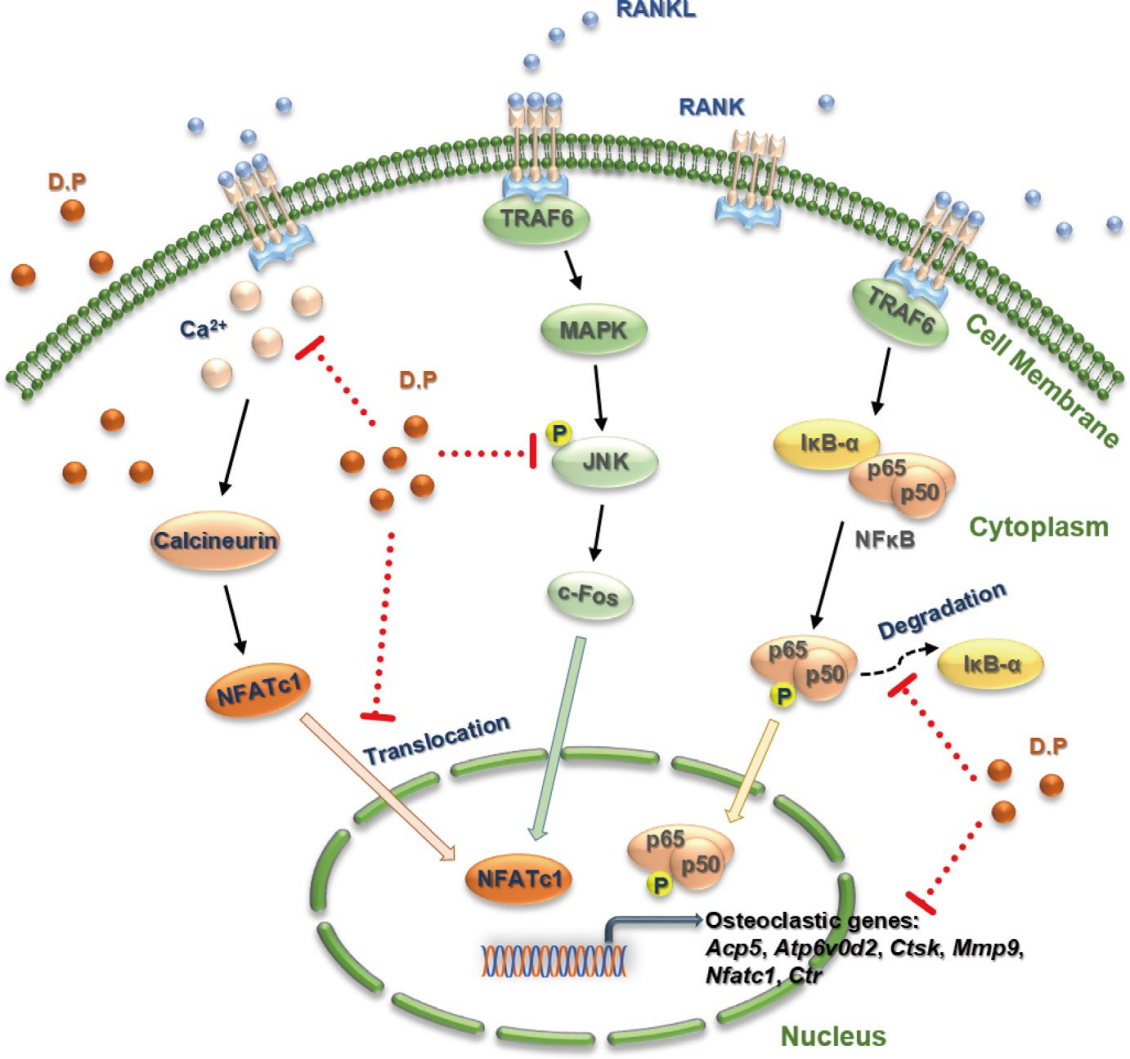
Proposed model showing that mechanisms of D.P in the suppression of RANKL‐activated JNK, NF‐κB and NFATc1 signalling pathways

As the downstream of the JNK and NF‐κB pathways, NFATc1 is the key factor in the initiation of the transcription of osteoclastic genes.^32^, ^35^ We demonstrated that both the increase in the gene and protein levels of NFATc1 and the trans‐nucleus activity of NFATc1 were inhibited by D.P. In addition, NFAT signalling pathway activates the PLCγ and leads to an increase in the intracellular Ca^2+^ level, resulting in calcineurin protein activation and downstream auto‐amplification of NFATc1.^36^ It was shown by the calcium oscillation assay that the activation of Ca^2+^ pathway by RANKL was inhibited by D.P. The reduction of the osteoclast‐specific genes determined by qPCR, was a result of the inhibition of NFATc1 activation by D.P. Therefore, we conclude that D.P inhibited the differentiation and function of osteoclasts by inhibiting the activation of NFATc1, which is a result of inhibiting the JNK pathway, NF‐κB signalling and Ca^2+^ signalling pathways (Figure 8). These in vitro results suggested the potential therapeutic effect against osteolytic diseases.

## CONFLICT OF INTERESTS

The authors declare no conflict of interests.

## AUTHOR CONTRIBUTIONS

In this study, Jiake Xu and Wei He designed and supervised the experiments. Yuhao Liu, Ziyi Wang, Chao Ma and Zhenquan Wei conducted most of the experiments, and Yuhao Liu did the statistical analysis. Kai Chen and Chao Wang participated in the fluorescence experiments. Chi Zhou, Leilei Chen, Qingwen Zhang and Zhenqiu Chen obtained the compound and other laboratory materials commercially, performed part of qPCR and Western blot assays. Ziyi Wang and Yuhao Liu wrote the manuscript. Jiake Xu, Yuhao Liu and Chao Ma revised the manuscript.

## Data Availability

The data that support the findings of this study are available from the corresponding author upon reasonable request.
